# Building trusting relationships while worrying about doing the wrong thing - a qualitative content analysis study on Swedish school nurses experiences of meeting students with trans experiences

**DOI:** 10.1186/s12912-025-03208-4

**Published:** 2025-05-15

**Authors:** Hanna Guvå, Marie Wilhsson, Margaretha Larsson, Lina Emmesjö

**Affiliations:** 1 School of Health Sciences, University of Skövde, Skövde, Sweden; 2 Institute of Health and Care Sciences, Gothenburg University, Box 100, Göteborg, 405 30 Sweden; 3 Halmstad’s Municipality, Halmstad, Sweden

**Keywords:** Nursing, School nurses, Transgender, Gender dysphoria

## Abstract

**Background:**

Young transgender persons who attend school are especially exposed to harassment, bullying, discrimination and violence in the school environment, experiencing an increased sense of mental and physical ill-health. School nurses work health promoting in schools and therefore have a unique opportunity to promote health among transgender students. There is however limited research on school nurses’ experiences of working with transgender students.

**Aim:**

To illuminate school nurses’ experiences of interacting with and supporting students with transgender experiences.

**Method:**

An inductive qualitative study with data collected through eight semi-structured interviews, analyzed through an inductive qualitative content analysis according to Graneheim and Lundman. The analysis was on the manifest level, where the steps were conducted in discussion within the research group to reach consensus through each step to ensure their connection to the aim. The analysis resulted in the findings, which is presented in two main categories with three sub-categories each.

**Findings:**

The school nurses supported the students with trans experiences through conversations during the health dialogues, building a trusting relationship with the students, and by being a spokesperson and for the students, with other students, teachers and parents. The school nurses also experienced uncertainty in which was the proper actions to support the students with transgender experiences and feared acting wrongly and therefore not supporting the students adequately or even harming students.

**Conclusions:**

The school nurses’ role in meeting these students is complex, where the school nurse work to build relationships with the students, but lack knowledge and tools. The lack of support and knowledge creates an ambivalence in how to best support these students, placing the school nurse before ethical dilemmas. The school nurses found support in the collaboration with the school counselor, as were the youth health clinics. School nurses should therefore, besides added education, be provided with arenas to discuss ethical dilemmas surrounding gender identity with other professionals who work in the school environment, or with adolescents.

**Supplementary Information:**

The online version contains supplementary material available at 10.1186/s12912-025-03208-4.

## Background

Transgender persons experience that their gender does not align with the one assigned at birth [1]. Young transgender persons rate their health to be average or poor twice as often, anxiety to be three times as common and a lower quality of life than other cisgender people [2]. Young transgender persons who attend school have been expressed in reports to be especially exposed to harassment, bullying, discrimination and violence in the school environment, which has been connected to an increased sense of mental and physical ill-health [3–8], leading to school absence and lowered school results than cisgender students [8]. Promoting inclusive and safe school environments has been argued to lower the risk of discrimination and bullying for transgender students, significant for transgender students’ health [9], where school nurses have a pivotal role in creating such an environment. The school nurse carries the medical responsibility in the student health organization, by of lessening the risk factors for ill-health and improving the protective factors for the students in Sweden [10]. School nurses have been reasoned to have a unique opportunity to promote health among transgender students through encouraging norm criticism in the school environment, which examines how different norms influence peoples everyday life and values in society [1]. School nurses can also contribute to a safe inclusive school environment [11–14].

There are a limited number of studies which explore students with trans experiences views on the school nurses, as well as school nurses’ own experiences of interacting and supporting students with trans experiences. The studies found focus broadly on LGBTQ (lesbian, gay, bisexual, trans and queer) students in relation to the school nurse [12, 15], on the school environment as a whole [8, 16] or combine school nurses and physicians experiences [17]. One American study focus on the perspective of the school nurses’ work with supporting students with trans experience [18]. The studies have been conducted by two research groups, one in Finland [12, 15] and one in the state Minnesota in America [17, 18]. In the few studies conducted, both students and school nurses expressed that school nurses lack knowledge in how to meet and support LGBTQ students [12, 15]. School nurses are expressed to lack knowledge and education, feeling insecure in their professional role [15]. Continuous education for school nurses on transgender topics has been expressed as paramount for improving health for transgender students [8, 16–18]. Because of the limited material on specifically school nurses’ experiences of interacting and supporting specifically students with trans experiences, additional research is needed to build knowledge on the phenomenon. Students with trans experiences have greater mental and physical ill-health, the school nurses have a unique role since they interact with the students several times during adolescence, as well as having the responsibility to work health promoting with students. Capturing the school nurses’ experiences is therefore paramount in building knowledge on how to improve health for adolescents with trans experiences. The aim was therefore to illuminate school nurses’ experiences of interacting with and supporting students with transgender experiences.

## Method

An inductive qualitative study design where data was collected through semi-structured interviews with eight school nurses in Sweden. The inductive approach is data driven [19, 20] and characterized by the search of patterns [20]. This was appropriate when answering the study aim, since it allowed for capturing the school nurses specific experiences and through analysis moving towards a larger whole [19]. The inductive approach is recommended when there is a lack of former knowledge on a phenomenon [19], which the phenomenon of school nurses interacting and supporting students with trans experiences is. The interviews were conducted with the support of an interview guide, which allowed for accessing the school nurses’ experiences of the phenomenon, which was appropriate to answer the aim. The data collected were audio recorded, transcribed into text, and analyzed through qualitative content analysis on the manifest level into categories and sub-categories [21]. The choice of analysis method allowed for a descriptive and close-to-text analysis of the school nurses experiences.

### Setting

This study was conducted in Sweden, where the student health organization at are required by law [22] to work with health promotion to contribute to the students’ health and learning, and to prevent illness and remove obstacles of learning and development [23]. The school nurses invite students to four health dialogues with the students throughout their years in school. The health dialogues have their base in a health form which each municipality or region creates, and include subjects such as school situation, relationships, physical activity, meals, tobacco use, alcohol, narcotics, sexuality and health. The health forms sometimes include for the students to choose their gender identity, but it is rare for there to be another option than male or female. he school nurse carries the responsibility of lessening the risk factors for ill-health and improving the protective factors for the students [t^10^].

### Recruitment and participants

Inclusion criteria for participation were school nurses who were working at middle- and high schools, having worked as a school nurse in such a setting for at least one year. Information letters and a request for permission to conduct interviews with school nurses were sent to principals in one municipality in the south of Sweden. The request did not receive any response. Instead, the researchers reached out to two school nurses in the municipality by email, who were informed of the project through an information letter and were asked if they wanted to participate. Both school nurses agreed and asked their principals if they were allowed to participate. Both principals agreed. Because of the difficulties in recruiting from the principals, snowball sampling was used to recruit participants, which was successful in recruiting participants. The participating school nurses were asked if they knew someone else who worked as a school nurse who they could provide contact information to. The researchers then contacted the suggested school nurses and asked if they wanted to participate. The school nurses who agreed asked their principal if they could, and all agreed. Snowball sampling uses the network of the participants, who recommends further possible participants. It is especially adventurous when collecting data with participants which may be difficult to get in touch with [24]. The first school nurses recommended two possible participants each, two who accepted and two who declined. The process was repeated until eight school nurses were interviewed (Table 1), all who were female.

**Table 1:** Participants age, work-life experience and masters

	Participating school nurses
Age range (y)	36–59
Range of Work-life experience as a school nurse (y)	2–18
Master in school nursing (n)	1
Master in midwifery (n)	1
Master in pediatric nursing (n)	2
Masters in primary health care nursing (n)	4
Total (n)	8

The snowball sampling led to a geographic variety among the participants, where school nurses who worked in the southwest of Sweden participated. The municipalities varied in size from above half a million to 26 000 citizens. Seven of the participants were working at high schools, and one at middle school. However, several of the school nurses working at high schools had previous experience of working as school nurses at middle schools. The participating school nurses had an even distribution of working for private and municipality run schools.

### Data collection

Data was collected through semi-structured interviews using an interview guide (included as a supplementary file). The interview guide was created after a literary search on previous research on the phenomenon, seen in the background section. The literary search informed the discussion within the research group of subjects to include in the interview guide. All researchers had experience in creating interview guides for semi-structure interviews, conducting semi-structured interviews, and analyzing such material. The subject, formulation of questions and question order were discussed in the research group until consensus was reached. The interview guide was then tested in two pilot interviews, on two school nurses. The audio recorded interviews were then brought back to the research group, which listen to them and deemed the interview guide sufficient to collect data to answer the study aim. The pilot interviews were therefore used in the data collection as well.

Initially, demographic data concerning the school nurses’ age, gender, education, current workplace and work-life experience was collected at the start of the meeting with the school nurses. Then, questions were asked about the school nurses’ previous knowledge on transgender persons and possible previous education on transgender persons to gain an understanding of the school nurses pre-understanding. This section started with the question ‘What do you know about transgender identity, gender identity, various gender identities, and gender dysphoria?’ The semi structured interviews question included open-ended questions for the school nurses to provide their experiences. The main interview started with the question ‘What is your experience with encountering students who identify as transgender or have a gender identity other than male/female (non-binary)?’ The interview questions then on the school nurses’ experiences of meeting students with transgender experience. Follow up questions were asked which allowed for participants to deepen their descriptions of their experiences, such as ‘did you find anything particularly difficult or easy?’, ‘did you experience any ethical dilemmas’ and ‘would you approach the situation differently today?’. The interviews were voice-recorded on an audio recorded. Seven interviews were conducted digitally, through an online based video communication tool. One interview was conducted in person. The participants chose the location of the interviews, which included if the interview was performed in person or digitally. The interviews lasted between 30–60 minutes.

### Data analysis

The data was analyzed through qualitative content analysis [21], which is appropriate when the goal is to provide knowledge and new insights of a phenomenon. The purpose is to attain a condensed as well as broad description of the findings in concepts, categories and themes [25]. The manifest content was prioritized, which is the descriptive content of the text. Categories are internally homogenous, and externally heterogeneous [21]. The interviews were transcribed manually right after the interview into text but were listened to and read several times to gain a sense of the overall content of the data. After this meaning units which addressed the aim were drawn from the transcribed text. The meaning units were copied from the interview text into a separate file into a table with the headings: *meaning units*, *condensation*, and *code*. The connection between the aim and the meaning units copied into the table were discussed between the H.G, M.W and L.E. When consensus was reached, the meaning units were abstracted and condensed to capture the meaning of the meaning units by H.G & M.W. The condensed meaning units were then discussed between H.G, M.W and L.E, and written into a code which illustrated the core of the meaning unit. The codes were then sorted into content areas through discussions to reach consensus within the research team to ensure rigor and trustworthiness. The codes were then analyzed into sub-categories, which were named through a discussion between H.G, M.W and L.E. The sub-categories were grouped together into categories, which were named in the same manner (Table 2) on the manifest level. The transparency of the analysis is visible in the findings through quotations, which also illustrate the results. The analysis process was conducted through a back-and-forth process in the whole research group, with transparency between all co-authors.

**Table 2:** Excerpts of the qualitative content analysis processes

Meaning unit	Condensation	Code	Sub-category	Category
*“First of all*,* it’s very sensitive*,* it’s very … like you have to be very soft*,* and like try to listen in where they are*,* what they want to talk about”*	Its sensitive, there is a need to be soft, listening in	Intuitive/listening in	Building trusting relationships to enable for students to open up	Enabling interactions which support students with transgender experience
“*I think that becoming a young adult*,* moving from being a child to an adult*,* it’s an identity crisis for everyone. Regardless if you feel like you’re in the right gender. I think it’s anxiety-inducing for some to get breasts*,* pubic hair*,* and that it creates a general sense of anxiety and identity crisis*”	Puberty is an identity crisis for students, it is difficult to know if their experience is gender dysphoria or puberty	Difficulties differencing puberty and gender dysphoria	Ambivalence in the difference between puberty and gender dysphoria	Feelings of uncertainty when supporting students with transgender experience

### Ethical considerations

The study was conducted in accordance with the four ethical principles of the Declaration of Helsinki [26]; following the principles of autonomy, beneficence, non-maleficence, and justice. The study was designed, planned, and performed as per Swedish law [27], stating how ethical approval is not needed when persons are invited to participate voluntarily. Through the information letter, the participants were informed of the study aim, the data collection method, the responsible organization of the study as well as the confidentiality of their identity. The voluntary nature of participation was emphasized both in the information letter and orally. Informed consent was obtained both in writing and verbally. The participants were emailed the consent forms and sent them back by mail. Orally, informed consent was obtained prior to the start of the interview. Some of the data was collected in relatively small municipalities, in a defined geographical area, which was considered when presenting the characteristics of the school nurses in the method section as well as the findings of this study. The participating school nurses are therefore not defined individually, but instead as a group. Data was kept on password protected servers, which only the research group had access to. Only H.G had access to the identity of the participants, to ensure the confidentiality of the school nurses. In this case, regarding the school nurses’ experiences of meeting students with transgender experience were not considered vulnerable in the interview situation, since they were interviewed by a master student in school nursing and there was no hierarchical relationship between them and the one interviewing them. However, participating in an interview about a topic which may be considered sensitive could have been difficult, tiring and burdensome for the participating school nurses, even if it was not their gender identity which were discussed. These factors were discussed in the research group prior to the data collection and were considered when planning the time and setting for the data collection with the school nurses. A good amount of time was allotted for each interview, and the school nurses chose the time and place for the interview to ensure they felt comfortable. No participant was perceived to have experienced discomfort during the data collection.

### Findings

The findings are presented in two main categories: *Enabling interacting which support students with transgender experiences* and *Feelings of uncertainty when supporting students with trans experience*. Each of the main categories have three sub-categories (Table 3).

**Table 3:** Description of finding structure

Main categories	Subcategories
Enabling interactions which support students with transgender experience	• Health dialogues enabling conversations about gender identity • Building trusting relationships to enable for students to open up • Being a representative for the student
Feelings of uncertainty when supporting students with transgender experience	• Ambivalence in the difference between puberty and gender dysphoria • Worry of supporting students in the wrong way • Fear of acting wrongly in their professional role

### Enabling interactions which support students with transgender experience

The personal meeting between the student and the school nurse created the arena for where conversations about gender identity and trans experiences could transpire. The health dialogues which the school nurse had with each of the students created a gateway for deeper conversations. The school nurses argued for the importance of building relationships and creating safe spaces relied on trust and respect. The role of the school nurse also required them to be the spokesperson for the student towards others, where conversations were the arena for meeting others of different minds.

### Health dialogues enabling conversations about gender identity

The school nurses experienced that meeting students with transgender experiences was benefited by the health dialogues which they invited the students to. Gender identity and gender dysphoria was brought up by the students when speaking on subjects such as sexuality, sexual identity, queer identity, relationships, body and gender development as well as norms about gender identity. Sexual health was expressed by the school nurses to always be discussed during the health dialogues, but to varying depth. One school nurse said: “I*t’s difficult to know how deeply to address these questions. I’m just a school nurse after all*” (Participant 8). Another school nurse expressed: “*I’m address the subject early*,* about how they feel about their body*,* if they feel like they’re in the right body*” (Participant 3). There was a shared view among the school nurses that students rarely brought the subject of gender identity, gender dysphoria or being trans during the health meetings. However, the health dialogues were important for enable a connection with the students for future conversations.

Before the health dialogues, the students filled in a questionnaire which was the base of the conversation during the health meeting, expressed to be valuable in the conversations with the students. The questionnaires were different in different regions, where some held more in-depth questions about sexual health and gender, and others had more overarching questions. One school nurse said: “*It would be good to add it*,* sometimes they [the students] add things*,* and then you can talk about it. It would be better just to add it to the form*,* about gender*,* because then there’s an opening about it. It’s relevant*,* because then we can help them*”(Participant 2). The school nurses had differences in experience in speaking to the students about gender identity. One school nurse described how a student had added questions about gender identity on the questionnaire, illustrating how the health dialogue was a natural start of a conversation about gender identity. One school nurse said: “*I tell them [the student] that if there’s something [outside of the form] they want to discuss [during the health dialogues] we can*,* or I move on to the next question on the form*” (Participant 3). Even students not wanting to answer specific questions during the health dialogs was a part of enabling conversations, since then the school nurses knew what subjects were sensitive.

### Building trusting relationships to enable for students to open up

By using their conversation skills, the school nurses’ interactions with students with transgender experiences built trusting relationships. In the interaction with the students, the school nurses used their experience and intuition to get a sense of if the student felt weighed down by something. The school nurses’ experience was that the students rarely came to them to specifically speak about gender identity. One said: “*They come [to the school nurse]*,* not to talk about that*,* like not specifically that. There are other things which they come to speak to me about. It’s later*,* when we know each other that it comes out*” (Participant 4). Having conversations about other topics resulted in the building of a trusting relationship which enables students to open up. The students were often afraid to express their thoughts and feelings about feeling uncomfortable in their own bodies with anyone, according to the school nurses. In the trusting relationship, the school nurse could therefore support the students to become more comfortable in talking about their transgender experiences.

Building the relationship meant knowing the importance of moving carefully, daring to ask the difficult questions, but also to back off when it was needed. One school nurse said: “*First of all it’s very sensitive [talking about transgender experiences]*,* you have to be soft*,* try to get a sense of where they [the student] are. What they want to talk about*,* and never make them feel guilty*” (Participant 6). Different strategies were used by the school nurses, such as scheduling new meetings with the students to meet them several times to build the relationship, especially if the school nurse perceived the student to have difficulty to speak about their gender identity questions. One school nurse said: “*I might say*,* I’m here tomorrow if there’s anything you’re thinking about*,* I have an opening around noon. I won’t book something then*,* so if you feel like coming by you can. If you don’t want to*,* I have other things I can do too*” (Participant 7). The school nurses experienced enabling building trusting relationships by being supportive and confirming the students’ feelings where the students had started opening up and talking about their thoughts about their gender identity. Some students needed a trusting relationship with the school nurse, and the conversations it to understand themselves. Some students had not understood what was troubling them, but worried that there was something wrong with them. The school nurses expressed that it was important to normalize, explain, and encourage the students to try different gender expressions to see what feels right. One said: “*In the beginning they ask ‘What’s wrong with me?’ Then I tell them that there’s nothing wrong with you. Trying to normalize and explain that they’re born in the wrong body*,* and that they’re not the first one. It’s okay*” (Participant 3). In the trusting relationship, the school nurses could support the students’ experiences, while normalizing their feelings.

### Being a representative for the student

The school nurses meeting students with transgender experiences expressed becoming the spokesperson for the students with transgender experiences, which was at times lonely. The school nurses who met students with transgender experiences feared that the students with transgender experience would be exposed to bullying or discrimination, which made them worry over the mental well-being of the students. One said: “*The most important thing is that they’re okay. That they’re accepted. The problem isn’t that they’re in the wrong gender. The problem is what everyone else will think*,* that it’s taboo and embarrassing*,* a little unacceptable and unnormal*” (Participant 3). The school nurses educated classmates, teachers and other adults about transgender experiences. The school nurses experienced it as an unspoken expectation that they in their profession have knowledge to handle questions about LGBTQ identities, including transgender experiences and identity development, which made the teachers ask the school nurse for advice about conflicts and dilemmas surrounding identities. One school nurse said: “*One teacher was worried about gym class. Should the student change with the boys or girls? The student didn’t identify as either. We solved it by letting the student change in the teacher’s changing room*” (Participant 7). At times the school nurses also had to be the spokesperson for the student with their parents. Some students were expressed not to dare to tell their parents about their transgender experience, and some had parents who did not accept it. The school nurses expressed having the ability to create spaces for conversations, both for the student and the parents. The school nurses expressed to be seen by parents and the student as knowledgeable, but not in a power-position such as the teachers were. One said: “*One student really wanted me to participate in the conversation where he was going to tell his mom. So we had a little meeting at school. He was really worried that she wouldn’t understand. A fear that she would back up*,* but she was really understanding and explained that it didn’t matter”* (Participant 1). The school nurse therefore became a spokesperson both in the school, and outside of it with some students’ parents.

The school nurses saw their role as the first line of care for the students with transgender experience. The responsibility of the school nurses was not to know everything right away, but be a spokesperson by acquiring the needed knowledge, to follow up unanswered questions and to know where to direct those who they themselves cannot or should not help. The school nurses were adamant about their role in guiding the students towards the right type of health care. One said: “*That’s the entire school nurse profession. You’re like a platform*,* trying to help the students. I don’t have to be able to answer everything*” (Participant 6). The school nurses were therefore both a spokesperson and a guide in the students’ journey of exploring their gender identity.

Being the spokesperson for the students with transgender experiences was occasionally lonely and an exposed position to be in according to the school nurses. However, the school counselor was a close ally in this work, where some had close collaborations. One said: “*I think a close collaboration between the school nurse and school counselor is really important. It’s both. You need to see things from different perspectives*” (Participant 8). The youth health clinic was also expressed to be a good collaboration partner in the work with students with trans experience, having joined educational days and reflections. The collaboration and support of others aided the school nurses in being the spokesperson for the students, creating a sense of support for the school nurses.

### Feelings of uncertainty when supporting students with transgender experience

The school nurses who met students with transgender experiences felt uncertainty in what to do when they met of students with transgender experience. The school nurses described feeling ambivalence, worry and fear. The school nurses expressed a strong sense of care for the students and saw great suffering in the students with transgender experiences and identified needs which were rarely met. They also expressed ambivalence in if the expressed transgender experiences were truly gender dysphoria. The lack of evidence-based and clear guidelines in combination with the at times sharp debate on trans persons in the media created a sense of insecurity among the school nurses on the right actions to support the students. There was an overarching worry and fear of aching wrongly and therefore not supporting the students with transgender experiences adequately.

### Ambivalence in the difference between puberty and gender dysphoria

The school nurses’ supporting students with transgender experiences reflected on the reasons for some students experiencing gender dysphoria. There was an experience among the school nurses of an increase of students who questioned their gender identity and who had gender dysphoria. One of the school nurses said: “*We’ve talked about how there’s been an increase. We don’t have a lot of them here*,* but there are several students who struggle with this*” (Participant 4). Several of the school nurses expressed insecurity about whether the gender dysphoria was a part of the adolescent forming of the identity. Being uncomfortable during puberty was expressed by the school nurses to be common among adolescents in the process of becoming adults. They argued that all adolescents needed support in understanding their emotional reactions to puberty, which could make the adolescents feel better about the development of their identity. One school nurse said: “*I think that becoming a young adult*,* moving from being a child to an adult*,* it’s an identity crisis for everyone. Regardless if you feel like you’re in the right gender. I think it’s anxiety-inducing for some to get breasts*,* pubic hair*,* and that it creates a general sense of anxiety and identity crisis*” (Participant 2). There were other experiences among the school nurses as well, of a society becoming more informed and open, which allowed for reflection and conversations about gender, body and identity. They expressed how questions about gender identity had always been there, but that it was ignored previously. One said: *“I think a lot of students [historically] walked around with the same thoughts. There’s always been the same type of thoughts about it [gender identity]*,* but you didn’t know where to turn with such questions. It’s impossible to know if it has increased”* (Participant 7). There was therefore ambivalence within the school nurses on the students if the transgender experiences were gender dysphoria which needed diagnosis and care or merely stages of puberty.

### Worry of supporting the students in the wrong way

The school nurses who met students with transgender experiences worried that their support may encourage the student to make choices which they would regret later in life. They emphasized the importance of the students not making rash or impulsive decisions. There was a general worry among the school nurses of the students not being mature enough to make life-altering decisions, since their ability to understand consequences had not formed yet. One said: “*When it comes to gender*,* I’m expected to encourage everything that the teen feels. I don’t know. It troubles me. I think it’s ethically difficult to step in and encourage something which I may not think is the actual problem. Or maybe won’t be a problem in the future*” (Participant 5). Being explicit with the possibility for the student to change their mind was important according to the school nurses when they encouraged the students to try different types of gender expressions. Encouraging the students to take time and reflect was weighed against the sense of frustration and helplessness over the long waiting times in the specialist care. The school nurses expressed how the students who wanted to receive care often had to wait a long time. One school nurse said: “*I think they should be helped and supported quickly*,* that it takes so long is really difficult … they should be provided with help and support*,* with the right person to talk to*” (Participants 1). The school nurses were challenged by weighing the worry of the students changing their mind, against the long waiting time the students suffered by not seeking specialist care.

### Fear of acting wrongly in their professional role

The school nurses expressed the importance of being correct, respectful, and using the right pronoun and name for the students with transgender experience. They expressed that as the school nurse, they had gained a special confidence, and then they had the responsibility to act correctly. One said: *“You absolutely don’t want to be someone who says the wrong thing*,* especially as the school nurse. A school nurse is supposed to know*,* and she’s supposed to think and do the right thing. You feel insecure*,* if maybe you don’t support enough or think the wrong thing”* (Participant 6). At the same time, it was difficult to change the way one spoke. The school nurses expressed uncertainty about new terms connected to gender identity. They had the intention of doing things correctly but expressed how they at times failed. The school nurses also expressed an insecurity of how and if to document about students with trans experience. One said: *“I don’t know if it’s ethically right. Then it’s there*,* in black and white. What if they change their mind?”* (Participant 2). The school nurses were insecure about what was right according to laws and regulations as the students with transgender experiences were moving between gender identities, since there were gender differences concerning health programs in the school, such as vaccines. One school nurse said: “*I don’t want to do the wrong thing*,* but are they supposed to get the vaccines for girls or boys? I don’t want to refer my insecurity about what to do on the students during the transitioning phase*” (Participant 6). There was a fear among the school nurses of doing wrongly, both in the use of terminology, documentation, and in use of the regulations in the gender binary school health system.

## Discussion

In the findings, the conversation emerged as the school nurses’ most important arena and tool in the meeting with students with trans experience. The importance of building trusting relationships with the students was expressed by the school nurses in the study. This is similar to previous studies as well [28–31], where building trust has been expressed as essential to be able to help students in general as a school nurse [31]. Previous studies have also expressed how school nurses need to be able to speak about difficult subjects [30], which the school nurses in this study expressed as well. However, the school nurses in the current study were of different opinions if they should be the ones to breach the toping, or if it should be the students, illuminating a difference in their view of their role. The findings shows that the school nurses’ approach sensitive subjects by using experience, conversation skills, and intuition when they suspect that a student is carrying something sensitive. Similar findings has been argued for in past research [30], when school nurses try to creating a safe environment when discussing sensitive topics such as sexual health. The school nurses developed creative methods to approach sensitive topics, building trust to get students to open up about difficult topics. Similarly, previous research [30] argues for the importance of taking care of opportunities as they arise. As an example, in the clinical setting, it is of importance that the school nurse ask the student to sit and talk for a while when the student visits the school nurse. Capturing moments with students is crucial to continue to build trusting relationships when supporting students with transgender experiences.

The health dialogue was described as an opportunity to lay the foundation of the school nurse and student interaction. The health dialogue has been described in previous research as one of the school nurses’ most important health-promoting work tasks, with opportunities to discover physical and mental health issues, vulnerability and preventing risk factors [32, 33], which transgender students are known to have in adolescence [2, 7, 34] and in adulthood [35–37]. Supporting students with transgender experiences is therefore of high relevance in the school nurses’ role. In the findings, the health dialogue was described as a possible arena for conversations about gender identity and transgender experience. There were however variations in if the school nurses asked questions about gender identity or more general questions in sexual health during the health dialogues. There were differences in the design of the health forms and the inclusion of questions concerning sexual health and identity. Previous research [30] highlight the advantages of structured ask questions about sexual health and identity, partly to confirm that the subject is welcome and natural to talk about, partly so as not to miss the underlying causes of mental illness. The attitude to how intimate and explicit questions the school nurse should address is current varied, which was seen in this study as well. However, not approaching these questions can be explained by uncertainty related to lack of knowledge, experience and fear of doing wrong [17], or how transpersons have been observed to be stigmatized by in health care [38]. The stigmatization is often based on a lack of knowledge and stereotypes [39], which can be changes by an intersectional and norm-aware perspective, where one’s own preconceptions are illuminated [40]. It appeared however, that conversations about gender identity or sexual identity/orientation rarely arose during the health dialogues. However, the school nurses believed that the health dialogues could function as relationship building for further conversations of a more sensitive nature. Considering the findings of this study, and the presented previous research on transgender persons health, supporting students with transgender experiences is of great importance to fulfill the role of the school nurse. Including a question about gender identity in the health form is a structured and easy way which could help the school nurses to approach the subject and to start building that crucial trusting relationship.

The school nurses’ viewed their role as both the students’ spokesperson and as a platform for further referral to the right care, supporting previous research [18, 33], where the school nurses wanted to be a listening partner, but knew their organizations’ limitations within the school environment. This was seen both in the study on school nurse experiences of supporting transgender students [18], and school nurses experiences of working with students in general [33, 41]. The school nurse’s treatment and actions has been expressed in previous research to have a significant impact on the students feelings and well-being, with a key role in supporting students in the school environment [41]. The supporting role of the school nurse can therefore be seen as significant both in interacting with students in general but may be especially crucial when supporting students with transgender experiences. The school nurses should therefore be provided with the tools, such as an updated health form, and education in their general education and master education on gender identity to be able to perform their supporting role adequately. To ensure this, it should be included in the education program in the higher education law [42].

The findings revealed that the school nurses felt unsure of how to respond to the students with transgender experience, feeling afraid of accidentally behaving offensively. The school nurses expressed subtle and unwritten expectations from themselves and society, norms. Norms are expectations and values of what is desired in a society [1]. The discourse about what is considered normal has a strong influence on how society perceives and interprets norms and attitudes. As both young and transgender, the student breaks the normality template. Gender has been expressed as a social construct of what is performed through actions and expressions of normative expectations of gender [43], which the student challenges through questioning their gender identity. This places the school nurses in ethical dilemmas, since the Swedish school structure lacks tools to support and confirm transgender students through, among other things, an administration system that only allows two genders and classifies based on social security number and the students sex assigned at birth [44]. This was illustrated in the findings through the school nurses’ confusion on what vaccines the students should receive. The basic structure can be explained based on an intersectional perspective as a systematic injustice with limiting flexibility, where norms and injustices need to be brought to light [45], challenged and changed to create an inclusive environment. The school nurses are therefore left to sort through the structural impossibilities, without clear guidelines in how to do so, where the structural challenges are both on the national level with laws [46], and local level with a lack of school or municipality guidelines, illustrated in these findings. The school nurses should be provided with national support, and local guidelines, in what their role is and how to best support these students.

The school nurses’ experienced uncertainty in the challenges of students with transgender experience, with a strong sense of empathy, care as well as a persuasive willingness to help and support the students. However, the findings also showed that the school nurses were ambivalent about how students with transgender experience are best supported. There is, as presented, a lack of previous research on the phenomenon to explore the current study’s findings against. However, similar results were found in a study on school nurses’ experience of nursing work with students with mental illness [28], who also expressed worries in how to properly document the issues the students were having from fear of it burdening the students later in life. The school nurses in the findings also raised questions about whether gender dysphoria in adolescents is a part of puberty, and not related to a gender identity that does not correspond to the sex assigned at birth, placing the school nurses in an ethical dilemma. In the findings, the school nurses express having support from other professions, such as the school counselor. The support can help the school nurses to work through the dilemmas. Ethical reflection groups have been expressed to be of great health for school nurses when dealing with challenges in their work environment [47]. A need for structured reflection could aid the school nurses in how to support these students, which could be done with the school counselor, or other school nurses.

Adult society has historically a structurally demeaning view of young people’s ability to take responsibility [48], being irrational, immature and impulsive. The view reinforces normative ideas that identity issues are a developmental phase that passes and where the adult world must be steady and faithful [49]. This can be seen to enforce norms on gender for adolescents as well and influences the adolescents autonomy and own action power [50]. Instead, school nurses should apply an intersectional perspective, which illuminate power structures in health care and in the school environment [39]. In the findings, the school nurses expressed notions that gender dysphoria could be a symptom of other, underlying problems. There was concern among the school nurses that the students with gender dysphoria would regret it in the future if they made hasty and impulsive decisions that could affect the rest of their life. This has the risk of limiting the students’ autonomy, since the school nurse can an influential person in their school environment. However, the previous research does not give cause for this concern. There is currently no evidence found to show that adolescents are more prone to regret than adults [51]. Rather, previous research argues that a strong factor for ill-health is having to wait for gender-affirming care and that it is inhumane to force a young person to spend their adolescence suffering [52]. There is therefore a need for changes in legislation, education and locally to support school nurses, so they can face the challenges and opportunities in supporting students with transgender experiences.

### Methodological considerations

The trustworthiness of qualitative studies can be judged through the four criteria; credibility, dependability, transferability and confirmability [53]. Credibility is strengthened through a clear and transparent presentation of the participants [20], which was conducted in the method section. In regard to data collection, semi-structured interviews enable reciprocity between the researcher and participant [54], as well as allow for follow-up questions [55], which strengthened credibility of the findings and deepen them. Interviews were conducted both in-person and digitally. Video interviews increase the opportunity for long-distance participation [56, 57] which was seen in the present study. Digital interviews have been argued to be compatible with in-person interviews in terms of quality of data [58]. However, technical difficulties may arise which may influence the quality of the data during digital interviews [56], but were not experienced in the present study and did not influence the findings. In qualitative content analysis credibility is strengthened in how the categories have been analyzed on the manifest level, where credibility has been strengthened by the close-to-the-text way of working with the data throughout the analysis which is a significant part of in qualitative content analysis [21].

Dependability is described as a criterion for evaluating integrity in qualitative [59], made possible through the transparency of the research process [60]. Transferability is the extent to which the findings can be transferred to other settings or groups [59], made possible through the description of participants [20], the research context [53] and the analysis process. The snowball sampling provided participants with a range of ages, work-life experiences, master educations, and city size they worked in. However, since the possible participants were referred to by previous participants, there is a possibility that there was homogeneity in the experiences. The interviews with the school nurses illuminated a range of experiences, which created rich data to analyze, which were reached with the eight participating school nurses. Qualitative research exists in a paradigm of a value-based process, characterized by multiple realities and multifaceted perceptions of a phenomenon [20]. There was no goal of reaching saturation, rather a range in experience and to capture the school nurses’ descriptions were the objective. Confirmability is a criterion for the integrity of a study, referring to the neutrality of the data and interpretations [59], expressed to be established when credibility, dependability and transferability is achieved [53]. The intent is that the descriptions in the method section allow for the reader to consider the dependability, transferability and confirmability of the research findings.

## Conclusions

Swedish school nurses have experiences of meeting students with transgender experiences, students who define themselves as trans, as non-binary and students who experience strong ill health related to gender dysphoria. The school nurses’ role in meeting these students is complex, where the school nurse finds themselves with the skills of creating conversations and build trusting relationships with the students help them support the students. However, the school nurses also felt ambivalence, fear of doing wrong and met challenges in supporting students in the right way. Key findings were the school nurses’ dual role as advocates for the students in teaching other professions or parents, while still expressing a lack of knowledge themselves, which they could not show in their profession. Further research is needed to deepen the knowledge on school nurses’ emotional challenges, as well as practical strategies used to supporting transgender students, which this study has touched on but could be deepened with a clearer focus in future research aims.

There are also clinical implications of the findings:

- School nurses have skills in conversation and building relationships, which help them in supporting students with transgender experiences.
- School nurses experience ambivalence, worry and fear in acting wrongly, and express a need for knowledge, which could be provided with education on gender identities in their registered nurses and master’s in nursing education. To ensure this, it should be included in the education program in the higher education act.
- Local guidelines and ethical reflection groups on the school nurses’ role, and support on actions to take should be provided to aid school nurses in their work.

## Electronic supplementary material

Below is the link to the electronic supplementary material.

Supplementary Material 1

## Data Availability

The datasets analyzed during the current study are not publicly available due to ethical principles but data not comprising confidential information are available from the corresponding author on reasonable request.
