# Supplementary material for: Building trusting relationships while worrying about doing the wrong thing - a qualitative content analysis study on Swedish school nurses experiences of meeting students with trans experiences
Source: BMC Nurs. 2025 May 15;24:543. doi: 10.1186/s12912-025-03208-4 (PMC12083182; doi:10.1186/s12912-025-03208-4)
Supplement: Supplementary file 1 — Supplementary Material 1 [file 12912_2025_3208_MOESM1_ESM.docx]

# Interview guide

### Participant Information

- Gender
- Age
- Education
- Current workplace
- Years of experience as a school nurse
- Previous experience as a school nurse

### General Questions about Transgender Identity and Gender Dysphoria

- What do you know about transgender identity, gender identity, various gender identities, and gender dysphoria?
- Have you received any training/professional development/education related to LGBTQ+ topics? If yes, did the training include topics on gender identity, transgender issues, or gender dysphoria?
- Do you ask about sexual orientation or gender identity during health consultations?
- Are there questions related to LGBTQ+ issues or gender identity included in the health form?

### Personal Experience with Students who Have Transgender Identities or Gender Dysphoria

- What is your experience with encountering students who identify as transgender or have a gender identity other than male/female (non-binary)?
  - Possible follow up questions:
    - Did you find anything particularly difficult or easy?
    - Is there anything you think could have been done differently?
    - Would you approach the situation in the same way today?
- Have you observed an increase in students struggling with gender dysphoria?
  - Possible follow up questions:
    - If yes, what are your thoughts on this?
- What do you perceive as the needs of young people with gender dysphoria?
- What issues do they bring to the school nurse?
  - Possible follow up questions:
    - How do you assist them?
    - Do you experience any ethical dilemmas or challenges?
    - Are there any personal challenges?
    - What is your knowledge regarding treatment, approach, support measures, and collaboration for students with gender dysphoria?
